# Patient satisfaction, patients leaving hospital against medical advice and mortality in Italian university hospitals: a cross-sectional analysis

**DOI:** 10.1186/s12913-018-2846-y

**Published:** 2018-01-29

**Authors:** Tommaso Grillo Ruggieri, Paolo Berta, Anna Maria Murante, Sabina Nuti

**Affiliations:** 1 Management and Health Laboratory, Institute of Management, Scuola Superiore Sant’Anna di Pisa, Via San Zeno 2, 56127 Pisa, Italy; 2 Department of Statistics and Quantitative Methods, University Bicocca-Milan, Via Bicocca degli Arcimboldi 8, 20126 Milan, Italy

**Keywords:** Outcomes, Italy, University hospitals, Against medical advice, Patient satisfaction, Performance evaluation

## Abstract

**Background:**

Healthcare systems are increasingly focusing on outcomes that are the endpoints of care: patient health status and patient satisfaction. The availability of patient satisfaction (PS) data has encouraged research on its relationship with other outcomes, such as mortality. In Italy, an inter-regional performance evaluation system (IRPES) provides 13 regional healthcare systems with a multidimensional assessment of appropriateness, efficiency, financial sustainability, effectiveness, and equity. For university hospitals, IRPES includes the percentage of patients leaving hospital against medical advice (PLHAMA) and mortality rates at the ward level. This paper investigates the relationship between PS and PLHAMA across and within regional healthcare systems in Italy. Secondly, PLHAMA is used as a PS proxy to investigate its relationship with mortality at the ward level in the IRPES university hospitals.

**Methods:**

PLHAMA and mortality rates were gathered from administrative data, and PS scores from patient surveys. We explored the association between PS and PLHAMA through a correlation analysis, using data for the 13 IRPES regions. We tested this relationship also at the clinical directorate level in 28 hospitals in Tuscany (5482 interviewed patients in 100 clinical directorates). Secondly, we explored the association between PLHAMA and mortality at the ward level through correlation and regression analyses, using data of 405 wards of eight clinical specialties within 24 IRPES university hospitals.

**Results:**

Lower PLHAMA rates were associated with a higher PS in both regional and clinical directorate levels. A positive association between PLHAMA and mortality was shown at the ward level for IRPES university hospitals, with different results for medical and surgical clinical specialties.

**Conclusions:**

PS is an important performance dimension that provides healthcare managers and professionals with useful insights for improving care quality and effectiveness. Based on the study results, the PLHAMA rate could be regularly measured to highlight patient dissatisfaction. Due to the association between PLHAMA and mortality, this study also provides evidence of the importance of the patient perspective in assessing the quality of healthcare services. This relationship proved to be significant for surgical clinical units, suggesting the need for further analysing outcomes considering their different determinants in medical and surgical care.

## Background

Healthcare systems are increasingly measuring and comparing outcomes as the endpoints of care. Common outcomes measured within the clinical practice are mortality rates or survival and quality of life. However, when measuring quality of care, outcomes include both patient health status and patient satisfaction [1–4].

For this reason, patient perspective has been increasingly considered in performance evaluation in order to assess the results of healthcare systems and services.

Health organizations are therefore gathering and using patient-reported measures and including them in multidimensional performance evaluation systems beside measures based on other sources, such as administrative data [5–13].

The aim of this study was, firstly, to investigate whether a relationship exists between patient satisfaction (PS) and the phenomenon of patients leaving hospital against the medical advice (PLHAMA) in order to support the use of measures based on administrative data to assess PS with hospital services. Secondly, the study analyses the association between PLHAMA and mortality, which is a very commonly used outcome measure, using data at the ward level of 24 Italian university hospitals (UHs). This study also analyses the PLHAMA-mortality relationship across clinical specialties, in order to examine differences between inpatient pathways.

### Patient satisfaction and other outcomes

Patient-reported measures such as patient satisfaction, experience and perceived outcomes are widely collected and used to improve healthcare services [5–9, 11–13].

In the USA, the Hospital Consumer Assessment of Healthcare Providers survey assesses Medicare hospital quality through patient perspective. Twenty-five percent of the pay-for-performance program “Hospital Value-Based Purchasing” is based on these results. In the UK, patient perspective is considered as an important source of information to guide debate, patient choice, and research [7, 8, 14]. Several surveys are regularly conducted focusing on inpatient and outpatient services, emergency care, community mental care, maternity care, primary care, and social care. However, only a few providers use these data to promote improvement strategies [8].

In Italy, there are two significant systematic assessments of patient perspective. At a national level, the National Institute of Statistics (NIS) designs and administers a population survey every 5 years on patients’ satisfaction and their experience of access to health services. The institute provides results for each aggregate regional healthcare system [15]. At a regional level (Italy is divided into 20 regional administrations with 20 regional healthcare systems), the Management and Health Laboratory (Laboratorio Management e Sanità – MeSLab) of the Scuola Superiore Sant’Anna of Pisa conducts several systematic surveys for the Tuscan healthcare system on patient experience with hospital, emergency, primary, and maternity care services. The Regional Health Governmental Department, health authority CEOs and healthcare professionals use survey results to implement continuous improvements. At the regional level, data are used to compare and evaluate providers’ performance and to inform on the quality of healthcare services through a user perspective. Data are also used to create institutional accreditation indicators and to monitor the achievement of quality standards [16]. Health authority CEOs and professionals use these data to highlight strengths and weaknesses to be disseminated or improved respectively. In this sense, the providers’ budget system usually includes goals based on patient-reported measures. Through these actions and tools, healthcare policy-makers, managers and professionals thus activate quality improvement processes based on patient surveys’ results. In this regard, in the Tuscan context, it has been observed that hospitals report higher satisfaction scores when their professionals are more likely to know the results of patient satisfaction surveys [17].

The above mentioned experiences highlight how patient-reported measures can be used to improve healthcare services, despite the debate on whether patients are able to directly assess the quality of care they receive [18–22], and the resistance from both clinicians and managers in actually using these information and data [8].

The availability and use of patient satisfaction data have stimulated research investigating its relationship with other performance dimensions, such as clinical effectiveness, which refers to the recovery of functions or the survival achieved through healthcare treatments [23].

In this regard, research pointed out mixed results and conclusions [13, 18–22, 24–27]. For example, there is evidence on: i) a negative association between PS and inpatient mortality among patients with acute myocardial infarction [24] and in surgical care (where also hospital size and surgical volumes are important variables) [25]; ii) a positive relationship between PS with hospital care and quality of care with a focus on acute myocardial infarction, pneumonia, congestive heart failure and surgery [26]. On the contrary, other scholars have found that patients reporting a higher satisfaction (in particular, with physician communication) had higher healthcare expenditure, higher drug expenditure, lower emergency department and greater inpatient use, and a higher risk of dying [20]. Finally, a systematic review highlighted the importance of patient perspective in measuring quality of care, showing a positive relationship between patient experience, clinical safety and effectiveness [27].

To the best of our knowledge, evidence on the relationship between patient satisfaction and other outcomes such as mortality is lacking for Italy, although there is increasing availability of systematically collected data on patient satisfaction [11, 15] and other outcomes, in particular, from the National Outcome Evaluation Program [28].

### PLHAMA and PS

In Italy, as in other countries, patients can leave hospital or the emergency department without the approval of a physician by signing a document that removes the doctors’ legal responsibilities. Measuring this behaviour is important in order to evaluate the quality of care and to gauge patient dissatisfaction. Indeed, Hwang and colleagues found that dissatisfaction with treatment is one of the most commonly cited reasons for leaving hospital against medical advice [29]. Murante and colleagues observed that hospitals with a higher overall inpatient satisfaction and higher scores for patient-doctor relationship also have lower PLHAMA [11].

These results suggest that the PLHAMA indicator is able to capture the inability of healthcare services to meet patient needs. In fact, a negative hospital experience can affect patient behaviour so strongly that the patient may decide to leave hospital against medical advice [29]. Consequently, the PLHAMA rate may be used as an indirect measure of patient satisfaction.

### The context

In 2004, MeSLab developed a multidimensional performance evaluation system to compare and evaluate the results of the health authorities in Tuscany. Since 2008, an increasing number of Italian regional administrations voluntarily adopted the MeSLab performance evaluation system, creating an inter-regional network [30], which in 2015 included 13 regions: Basilicata, Calabria, Emilia Romagna, Friuli Venezia Giulia, Liguria, Lombardy, Marche, Autonomous Province of Bolzano, Autonomous Province of Trento, Tuscany, Puglia, Umbria and Veneto. The inter-regional performance evaluation system (IRPES) measures in benchmarking the regional healthcare performance, also across their 167 health authorities. The IRPES results are publicly reported on a specific website [31] and through a printed annual report [32]. The IRPES includes indicators for appropriateness, quality, efficiency, equity, integration and continuity of care, and financial sustainability. Only a few regions includes also results from surveys on staff satisfaction and patient satisfaction and experience in the hospital setting. In fact, surveys are time-consuming and they require additional human and financial resources. Surveys on patient experience (based on the same framework in order to ensure comparability) have been conducted in Tuscany (every 2 years since 2006), Friuli Venezia Giulia (in 2016) and Basilicata (in 2015–2016). In the IRPES, all the regions include two measures tracking patient behaviour, which are considered useful to estimate patient satisfaction and can be calculated through administrative data: the percentage of patients leaving hospital against medical advice (PLHAMA) and the percentage of patients leaving emergency departments without being visited or against medical advice.

In 2015, MeSLab included in the IRPES also a systematic assessment of hospital outcomes. To provide this evaluation, MeSLab adopted the indicators and the statistical methodology already developed in Lombardy by the CRISP (Centro di Ricerca Interuniversitario per i Servizi di Pubblica utilità), a research laboratory of the Bicocca University in Milan [33]. Thus, in 2015, MeSLab began to collect data on patient and ward characteristics from the IRPES regions in order to calculate and include in the IRPES risk-adjusted outcomes at the clinical specialty and ward levels, including PLHAMA and mortality rates.

### The study

The aim of this study was to investigate the relationship between patient satisfaction and PLHAMA in order to obtain evidence that supports the use of PLHAMA as a proxy of PS. We also explored whether PLHAMA is associated with mortality, in order to verify whether the patient evaluation can be aligned with other important and objective quality measures.

## Methods

To explore the association between patient satisfaction and PLHAMA, we used the Spearman correlation test and we analysed data from the 13 Italian regions joining IRPES. Patient satisfaction data were extracted from a national population survey administered in 2013 by the National Institute of Statistics. The patient satisfaction scores referred to the question: “With reference to your last hospitalization, to what extent are you satisfied? 0=totally unsatisfied, 10=very satisfied”. The 13 regional PLHAMA rates were based on the regional hospital administrative databases and extracted from the IRPES.

We tested the PLHAMA-PS association also within a regional context (Tuscany) at the clinical directorate level (i.e. organizational hospital aggregation of clinical specialties) by using the Spearman correlation test. For this analysis, we used data from Tuscany, the only IRPES region that, with the support of MeSLab, periodically conducts an inpatient experience and satisfaction survey with a sampling strategy at the clinical directorate level. Patient survey data (sample, *N* = 5482) referred to a two-month hospital discharge period in 2013 and 2014 and did not include patients hospitalized in an intensive care unit or for long-term care and patients died during or after the hospital admission. Interviews were conducted using computer-assisted telephone interviewing technique, in order to also reach groups of patients with a low literacy level [34]. The questionnaires covered important elements of patient experience and satisfaction, such as patient-doctor and patient-nurse relationships, communication, and overall evaluation of care. For our analysis, we considered the answers to the question “Overall, how do you rate the assistance you received in this hospital? From 0=very poor to 5=excellent”.

Secondly, we investigated the association between PLHAMA and mortality at the ward level using the CRISP-MeSLab database. The CRISP-MeSLab database included 2014 data for five surgical and three medical specialties from 24 IRPES university hospitals (UHs): general surgery, cardiac surgery, neurosurgery, orthopaedics-traumatology, urology, internal medicine, cardiology, and neurology. The database referred to the 2014 administrative data of the eight IRPES Regions that had participated in the CRISP-MeSLab outcome evaluation since 2015: Lombardy, Tuscany, Emilia Romagna, Friuli Venezia Giulia, Veneto, Liguria, Marche, and Umbria.

We chose to focus on the performance of the UHs to avoid the bias of possible confounders that could have been present by including different sized hospitals in the study: collinearity between high volume, hospital size and teaching status.

For each ward, we calculated the PLHAMA rates, defined as the number of discharges against medical advice divided by the overall hospital admissions, and the all-cause 30-day mortality rates, which included both inpatient mortality and mortality occurred within 30 days after the discharge. This second outcome was selected because it represents a relevant indicator to assess hospital care efficacy [35].

In order to exclude a potential bias linked to end-of-life paths (i.e. in the case of early discharges against medical advice when patients or their families prefer to receive end-of-life care at home), voluntary discharges followed by the patient’s death within 2 days were not considered in the PLHAMA rate.

For both the PLHAMA and the mortality rates, we only considered ordinary (longer than one-day) hospitalizations for patients over the age of 2 and residents in the regions where each university hospital is located. When a patient was admitted in one of the wards included in the analysis and then discharged by an intensive or a cardiac intensive care unit, mortality or PLHAMA that might have occurred were considered in the PLHAMA and mortality rates of the ward that admitted the patients. Finally, we included the wards with at least 100 discharges per year.

The CRISP-MeSLab database also included some variables, as illustrated in Table 1, to assess patient complexity. By including these variables in the analysis, we investigated the association between PLHAMA and mortality controlling for significant confounding factors.

**Table 1:** Patient complexity variables in CRISP-MeSLab database

Definition	Type	Criteria and Rationale
Gender	Dichotomous; 0 = Male, 1 = Female	Takes into account patient gender
Age	Continuous; > = 2	Takes into account patient age
Intensive Care passage	Dichotomous: 0 = No; 1 = Yes	Indicates whether the patient has been admitted or transferred in an intensive care unit (ICU) or a cardiac intensive care unit (CICU)
Sentinel event	Dichotomous: 0 = No sentinel event; 1 = At least one sentinel event	Identifies sentinel event/urgent case through 1051 specific ICD-9-CM codes
DRG weight	Continuous; > 0	Measures treatment complexity
Cardiovascular disease	Dichotomous: 0 = No; 1 = Yes	Indicates whether the patient was diagnosed with a cardiovascular disease during hospitalization through 386 ICD-9-CM diagnoses
Elixhauser comorbidity index	Discrete: From 0 to 6	Index of patient comorbidity at the admission time [39]

First, to test the association between the gross PLHAMA and mortality rates, we used the Spearman’s correlation test.

In order to further investigate the association between mortality and PLHAMA, a standard regression model was applied. This model was estimated in order to evaluate the relationship between gross mortality and PLHAMA rates at the ward level by adjusting for patient complexity with the variables illustrated in Table 1. The model thus included for each ward: i) the percentage of female patients; ii) the average patient comorbidity index, patient age, and DRG weight; iii) the percentage of patients admitted or transferred to an ICU/CICU, with a cardiovascular disease, or with a sentinel event/urgent case.

Three different log-log model strategies were applied with an increasing number of covariates. Firstly, a simple model (MODEL-1) was estimated, including patient/ward characteristics and the PLHAMA rate at the ward level. Secondly, eight fixed effects for the eight clinical specialties were included (MODEL-2). Finally, an interaction between the specialty fixed effects and the PLHAMA rate at the ward level was included (MODEL-3). The first model verified the significance of the relationship between mortality and PLHAMA adjusting for the patient/ward characteristics. With the second model, we controlled whether the association between mortality and PLHAMA was confirmed after the specialty fixed effect inclusion. Finally, we evaluated using MODEL-3 whether the relationship between PLHAMA and mortality was different over the specialties. In order to understand the effect of the interaction, we calculated the marginal effects that represent the expected change in mortality rate due to an increase in PLHAMA rate for each specialty included in the model. Data were processed using Stata Software, version 12.

Table 2 summarizes the methods, variables, databases and sources used in the study.

**Table 2:** Summary of research aims, methods, observations, databases and sources

Research aims	Variables	Test	Number of observations and level of analysis	Sources
Exploring the relationship between patient satisfaction and PLHAMA	PS and PLHAMA	Spearman correlation	- PS scores: sample of 60,000 households interviewed in Italian regions; IRPES regions (*N* = 13); - PLHAMA rate: IRPES regions (*N* = 13).	- PS scores: NIS population survey, year 2013; scale 0–10 from “totally unsatisfied” to “very satisfied”; - PLHAMA rates: IRPES data; hospital administrative database; year 2014.
	PS and PLHAMA	Spearman correlation	- PS scores: sample of 5482 patients interviewed in Tuscany; clinical directorates (*N* = 100) within hospitals in Tuscany (*N* = 28); - PLHAMA rate: approx. 4700 PLHAMAs out of approx. 520,000 discharged patients; clinical directorates (*N* = 100) within Tuscan hospitals (*N* = 28).	- PS scores: patient experience and satisfaction survey with hospital care; scale 0–5 from “Poor” to “Excellent”; year 2013–2014; - PLHAMA rate: MeSLab calculation; hospital administrative database; year 2013.
Exploring the relationship between PLHAMA and 30-day mortality, also across clinical speciality	Mortality and PLHAMA	Spearman correlation	Mortality and PLHAMA rates: approx. 2500 PLHAMAs and 19,300 deceased patients out of 350,000 discharges; wards (*N* = 405) of clinical specialties (*N* = 8) within IRPES university hospitals (*N* = 24).	CRISP-MeSLab database; administrative database; year 2014.
		Log-log regression model for mortality and PLHAMA rates with case-mix variables (Table 1) and clinical speciality fixed effects		

## Results

### PLHAMA and PS

The analysis performed across the 13 regions highlighted that regions with a higher PLHAMA rate registered lower overall patient satisfaction scores (*n* = 13, ρ = − 0.6419, *p* < 0.02). When we retested this association at the clinical directorate level using data for Tuscany, the results confirmed the negative relationship (*n* = 100, ρ = − 0.5102, *p* < 0.001).

### PLHAMA and mortality rates

The two investigation strategies (Spearman’s correlation and the regression model) highlighted a positive and significant relationship between mortality and PLHAMA, in particular for most surgical specialties.

As shown in Table 3, the Spearman’s correlation for the whole set of wards showed a positive and significant relation between the two indicators (*p* < 0.01). This positive relation was also confirmed when we separately considered 229 surgical wards (*p* < 0.01). When separately testing for each specialty, the correlation between PLHAMA and mortality was positive and significant for orthopaedics-traumatology (*p* < 0.01), general surgery (*p* < 0.01) and neurosurgery (*p* < 0.05) wards.

**Table 3:** Spearman’s correlation for PLHAMA and mortality rates

Clinical specialties		Number of wards	Spearman’s ρ
All departments		405	0.421**
Surgical specialties		229	0.2691**
Medical specialties		176	0.1189
Surgical specialties	Cardiac surgery	21	0.2776
	Neurosurgery	24	0.4815*
	Urology	32	0.1207
	Orthopaedics-traumatology	51	0.4778**
	General surgery	101	0.3804**
Medical specialties	Cardiology	37	0.2883
	Neurology	33	0.0424
	Internal medicine	106	−0.156

**p < 0.01, *p < 0.05

Conversely, there were not significant associations both for the medical specialty group and for each medical specialty (cardiology, neurology and internal medicine).

Finally, the results of the regression models are shown in Table 4. The model that included only the patient/ward characteristics (MODEL-1) showed a positive and significant relationship between PLHAMA and mortality. In MODEL-2, we included the fixed effects of the specialities. The result concerning the PLHAMA coefficient did not change its statistical significance, although the magnitude decreased. In column MODEL-3, the interaction between PLHAMA and specialties was included.

**Table 4:** Results of the three regression model strategies

	MODEL-1	MODEL-2	MODEL-3
	Log (Mortality)	Log (Mortality)	Log (Mortality)
*Gender (% of females)*	0.0310	−2.481***	−2.638***
*Age*	0.0902***	0.0795***	0.0752***
*DRG weight*	0.285**	0.144	0.108
*% of sentinel events*	3.217***	1.969***	1.764***
*% of cardiovascular diseases*	−1.176***	−1.083*	−0.791
*% of passage in ICU/CICU*	−0.296	0.172	0.386
*Elixhauser Index*	0.862***	0.210	0.180
*Log(PLHAMA)*	0.257***	0.200***	0.00871
*Cardiac surgery*		0	0
*Cardiology*		−0.459	−0.374
*General surgery*		0.0951	0.910
*Internal medicine*		1.092*	1.622**
*Neurosurgery*		0.279	0.979
*Neurology*		0.578	1.060*
*Orthopaedics- traumatology*		−0.485	0.565
*Urology*		−1.635**	−1.167
*Cardiac surgery # Log(PLHAMA)*			0
*Cardiology # Log(PLHAMA)*			−0.124
*General surgery # Log(PLHAMA)*			0.331
*Internal medicine # Log(PLHAMA)*			−0.0329
*Neurosurgery # Log(PLHAMA)*			0.282
*Neurology # Log(PLHAMA)*			−0.0130
*Orthopaedics-traumatology # Log(PLHAMA)*			0.575**
*Urology # Log(PLHAMA)*			0.123
*Constant*	−5.579***	−3.253***	−3.496***
*Observations*	405	405	405
*BIC*	1157.7	1076.9	1075.8

* *p* < 0.05, ** *p* < 0.01, *** *p* < 0.001

This model allowed to investigate whether there was a different relationship between PLHAMA and mortality depending on the type of specialty. In order to analyse these results, we calculated the marginal effects of the interactions (Table 5).

**Table 5:** Marginal effects of the interactions included in the model

Clinical specialty	Marginal Effects (dy/dx)	Standard Error	Z	P > z	95% Conf. Interval	
Cardiac surgery	0.0087	0.1744	0.05	0.96	−0.334	0.351
Cardiology	−0.1152	0.1196	−0.96	0.336	−0.350	0.120
General surgery	0.3399	0.0665	5.11	0.000**	0.209	0.471
Internal medicine	−0.0241	0.0829	−0.29	0.771	−0.187	0.139
Neurosurgery	0.2905	0.1442	2.01	0.044*	0.007	0.574
Neurology	−0.0043	0.1150	−0.04	0.97	−0.230	0.222
Orthopaedics-traumatology	0.5833	0.0912	6.39	0.000**	0.404	0.763
Urology	0.1316	0.1301	1.01	0.311	−0.124	0.387

**p < 0,01; *p < 0,05

General surgery, neurosurgery and orthopaedics-traumatology presented a significant positive relationship between PLHAMA and mortality. Hence, there was an overall positive relationship in the models, as shown in columns MODEL-1 and MODEL-2 in Table 4, as well a specific relationship in some surgical departments. However, the same relationship was not observed in the medical wards.

## Discussion

In this study, we firstly investigated the relationship between patient satisfaction and the phenomenon of patients leaving hospital against medical advice. We also investigated the relationship between PLHAMA and mortality rates at clinical specialty and ward levels in 24 Italian university hospitals.

This study firstly pointed out a negative association between PS and PLHAMA. Based on this evidence, PLHAMA could be interpreted as the result of poor patient satisfaction with hospital care, as suggested by other scholars [11, 29], and thus PLHAMA can be used as a proxy measure to indirectly track PS.

Measures based on administrative data, such as the PLHAMA rate, can therefore be used by healthcare managers and professionals to assess PS in addition to other tools, such as questionnaires. As PLHAMA rate is based on administrative data, it can be easily and regularly monitored and compared across and within regions, hospitals and wards. We therefore recommend its inclusion in performance evaluation systems aimed at benchmarking healthcare systems and providers with a multidimensional perspective.

Secondly, we showed a positive relationship between PLHAMA rates, used as a proxy for PS, and mortality rates. Our results indicate that the phenomenon of PLHAMA, which is related to PS, is lower in wards that show better clinical effectiveness. This preliminary evidence from Italy suggests that there is no trade-off between the evaluation of healthcare services provided through the patient perspective and through measures of clinical effectiveness, such as mortality rates. Patients seem thus able to assess the quality of care they receive, as suggested by other authors [18, 21].

Healthcare managers and professionals should thus constantly monitor the PLHAMA rate as it can be used with a twofold aim: catching patient dissatisfaction with hospital services and warning of potential issues linked to other outcomes such as poor clinical effectiveness.

In addition, we showed evidence on this relationship also at the clinical speciality level. In particular, the study results were confirmed considering the services provided by surgical wards. The different results between surgical and medical inpatient activity may be rooted in different determinants of patient satisfaction [36] and the evolving role of hospitals, which is rapidly changing along with the epidemiological context [37]. The surgical inpatient care (especially in elective surgery) can be still considered as the “traditional” hospital acute care service, since surgical interventions are expected to be crucial for patient recovery and the most important care phases are expected to end with patient discharge. Conversely, in Italy, as in other countries, medical inpatient care is often a phase within a multi-provider chronic disease path, where continuity of care is essential to drive outcomes [38]. Hence, in medical care pathways, outcomes such as patient satisfaction may not just be related to the inpatient activity but to the overall care provided along the multi-provider healthcare service chain.

In surgical pathways, outcomes such as patient satisfaction may be related to hospital ward activities, such as respect for patient preferences through shared patient-doctor decision-making, the timeliness of surgery, and the health status during the post-operative phase. In these care paths, patients may be more capable of evaluating the quality and effectiveness of care, as most of the relevant care phases take place within the hospital itself. In medical pathways, this relationship may be more complex and merits a multi-provider perspective. Performance in medical care pathways should be increasingly evaluated considering a multi-provider, multi-professional and multi-disciplinary perspective, without focusing just on the individual hospital phase, because integration of care along these paths is crucial, in particular for patients with chronic diseases [38].

As suggested by this study, when interpreting and comparing results on patient satisfaction and other outcomes, researchers, healthcare managers and professionals should thus take into account the differences between surgical and medical multi-provider care paths.

This study has some limitations. Firstly, the results are limited to the clinical specialties included in the analysis. However, these units provide a high share of the overall ordinary hospitalizations in the IRPES UHs. For instance, these specialties provide the 50% of overall hospital discharges of the UHs in Tuscany. Secondly, the focus is limited to Italy. However, contextual factors are important elements in studies aimed at providing managerial and policy recommendations.

In order to improve results’ comparability among different regional and hospital contexts, we controlled for some potential distortions. Firstly, by excluding the PLHAMA of patients died within 2 days after discharge, we avoided the potential bias linked to patient end-of-life pathways. Furthermore, through the risk-adjustment provided in the regression model, we took into account the patient complexity of each ward. By excluding the patients living in a different region of the admitting hospital, we avoided the potential bias caused by patients leaving hospital against medical advice because they preferred to be admitted to a hospital nearer home. Finally, considering both inpatient and post-discharge mortality provided a more appropriate and precise estimate of the overall quality of care and effectiveness of the hospital services.

Further research will explore whether the evidence of this study is observable also for non-university hospitals and which are the factors that may contribute to the different associations between PS, PLHAMA and mortality in medical and surgical pathways.

## Conclusions

This study highlights the need to take into account patient satisfaction as an important performance dimension, which can provide healthcare managers and professionals with useful insights on how to improve care quality and effectiveness.

It is thus important to provide healthcare managers and professionals with measures that highlight issues in terms of PS, especially when patient surveys cannot be systematically administered.

This paper firstly provided evidence that the PLHAMA rate can be regularly used as a useful measure to highlight patient dissatisfaction, together with patient surveys, which can be periodically administered to fully explore the determinants of patient satisfaction and to disseminate best practices and manage weaknesses.

In addition, by testing the relationship between PLHAMA and mortality at the clinical specialty level, this study also aimed to contribute to the debate on whether and in which contexts patients are able to assess the effectiveness and quality of healthcare services [18–22].

Due to the observed association between PLHAMA and mortality rates, patient satisfaction merits increasing attention both as a specific goal of healthcare organisations and as a measure to catch potential issues in other performance dimensions, such as clinical effectiveness. Furthermore, the potential different determinants of outcomes in surgical and medical pathways should be considered when interpreting the results.

The increasing availability of published data and measures should encourage further studies on the association between patient satisfaction and other outcomes and the factors that contribute to this relationship. Research in this field can strengthen the significant role of patients in assessing the quality of care they receive.

## Abbreviations

CICU: Cardiac intensive care unit
CRISP: Centro di Ricerca Interuniversitario per i Servizi di Pubblica utilità
ICU: Intensive care unit
IRPES: Inter-regional performance evaluation system
MeSLab: Laboratorio Management e Sanità - Management and health laboratory
NIS: National Institute of Statistics
PLHAMA: Patients leaving hospital against medical advice
PS: Patient satisfaction
UH: University hospital
